# A highly conserved family of inactivated archaeal B family DNA polymerases

**DOI:** 10.1186/1745-6150-3-32

**Published:** 2008-08-06

**Authors:** Igor B Rogozin, Kira S Makarova, Youri I Pavlov, Eugene V Koonin

**Affiliations:** 1 National Center for Biotechnology Information NLM, National Institutes of Health, Bethesda, MD, 20894, USA; 2 Eppley Institute for Research in Cancer and Allied Diseases, Departments of Biochemistry and Molecular Biology, and Pathology and Microbiology, University of Nebraska Medical Center, Omaha, Nebraska, 68198-6805, USA

## Abstract

**Abstract:**

A widespread and highly conserved family of apparently inactivated derivatives of archaeal B-family DNA polymerases is described. Phylogenetic analysis shows that the inactivated forms comprise a distinct clade among archaeal B-family polymerases and that, within this clade, Euryarchaea and Crenarchaea are clearly separated from each other and from a small group of bacterial homologs. These findings are compatible with an ancient duplication of the DNA polymerase gene followed by inactivation and parallel loss in some of the lineages although contribution of horizontal gene transfer cannot be ruled out. The inactivated derivative of the archaeal DNA polymerase could form a complex with the active paralog and play a structural role in DNA replication.

**Reviewers:**

This article was reviewed by Purificacion Lopez-Garcia and Chris Ponting. For the full reviews, please go to the Reviewers' Reports section.

## Findings

DNA polymerases are enzymes that are essential for genome replication and repair in all cellular life forms [1,2]. There are several distinct families of DNA polymerases some of which are unrelated to each other whereas most show varying degrees of relationship [3,4]. All archaea and eukaryotes encode at least one but typically several paralogous B-family DNA polymerases that play a key role in DNA replication [5]. Euryarchaeota typically posses one or two B-family polymerase, in addition to the apparently unrelated D-family polymerase whereas Crenarchaeota have two or three paralogous B-family polymerases but no D-family polymerases [6-8].

**Figure 1:**
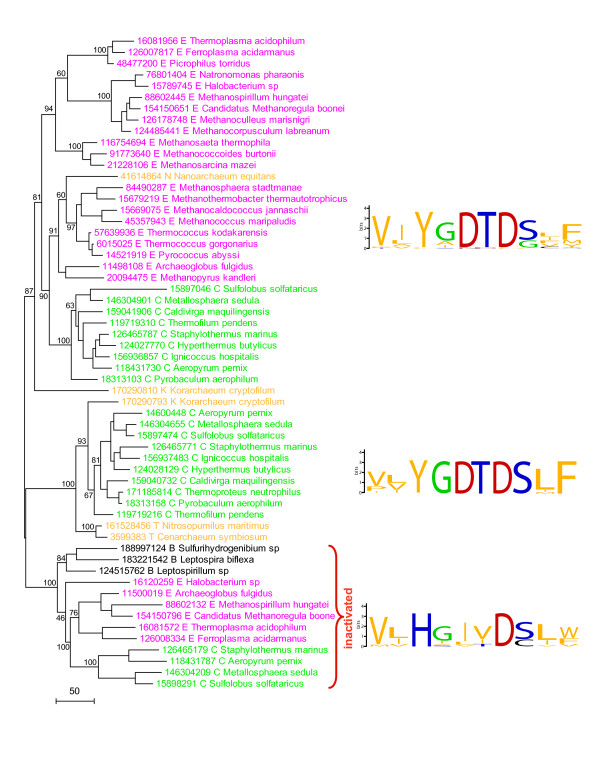
**Inactivated derivatives of B-family DNA polymerases in archaea: inactivation of the principal catalytic motif and phylogeny**. The right part of the figure shows the sequences of the essential Mg2+-binding motif of B-family DNA polymerases for two families of active archaeal polymerase (top and middle) and the inactivated derivative (bottom). The motifs are shown in the form of sequence LOGOs where the height of the amino acid symbols is a function of the frequency of the given amino acid in the given position [20,21]. The left part of the figure shows a phylogenetic tree for archaeal B-family DNA polymerases. Color code: purple, Euryarchaeota (E); green: Crenarchaeota (C); brown: organisms that might represent distinct arcaheal phyla: Nanoarchaeota (N), Korarchaeota (K), and Thaumoarchaeota (T); black, bacteria (B). Each organism is denoted by the full systematic name and the Gene Identifier (GI) number. Multiple sequence alignment of archaeal B-family polymerases (Additional File 1) was constructed using the MUSCLE program [22]. The tree was built using the maximum likelihood method implemented in the MOLPHY program [23] by local rearrangement of an original Fitch tree [24]. The same program was used to compute bootstrap values which are indicated (%) for selected major branches.

When examining the relationships between archaeal B-family DNA polymerases, we unexpectedly observed that in many Crenarchaeota and Euryarchaeota, one of the B-family DNA polymerase paralogs, despite the high level of overall sequence conservation, contains disrupted versions of the sequence motifs that are known to be essential for the catalytic functions. The polymerases of the B-family contain two enzymatic domain, namely, the N-terminal 3'-exonuclease domain that performs proofreading and the C-terminal polymerase proper domain [9,10]. In the apparently inactivated archaeal polymerase, the sequence motifs that in the active forms harbor the catalytic amino acids required for each of these activities are partially disrupted (Fig. 1 and Additional File 1). In particular, the crucial, most conserved YGDTD motif that coordinates Mg^2+ ^in B-family DNA polymerases and in which both aspartates and the tyrosine are essential for the polymerase activity [11-13] harbors at least two replacements in the apparently inactivated derivatives although it is notable that the distal aspartate is conserved and so is likely to retain an important function (Fig. 1). In addition, the most conserved DIE motif (Exo I) of the N-terminal 3'-exonuclease proofreading domain does not have a clear counterpart in the corresponding part of the polymerase derivatives described here (Additional File 1), strongly suggesting that the exonuclease activity is inactivated as well.

Phylogenetic analysis of archaeal B-family polymerases showed that the inactivated forms comprised a distinct clade with a 100% bootstrap support (Fig. 1). Moreover, within this clade, the Euryarchaeal and Crenarchaeal forms were clearly separated, also with full bootstrap support, and joined a third subclade that included similarly inactivated homologs from three diverse bacteria (Fig. 1). The presence of the inactivated DNA polymerases in diverse subsets of both Euryarchaeota and Crenarchaeota is compatible with an ancient duplication of the DNA polymerase gene followed by inactivation and parallel loss in some of the lineages. However, a relatively later duplication, inactivation and subsequent spread via horizontal gene transfer also could be a viable evolutionary scenario for the inactivated polymerases.

Inactivation of the enzymatic function of a protein and utilization of the inactivated protein for non-enzymatic (structural) roles, so that it retains substantial sequence conservation, is not without precedent. Perhaps, the cases that are most relevant for the present finding are the apparent inactivation of the small subunits of DNA polymerases and the N-terminal 3'-exonuclease domains in eukaryotes. In all Euryarchaeota, the small subunits of PolD contain all sequence signature of the calcineurine-like superfamily of phosphoesterases [14] and have been shown to possess 3'-exonuclease, proofreading activity [15-17]. By contrast, in the eukaryotic orthologs of these proteins, most of the catalytic residues are replaced, so the protein apparently performs a structural role in the polymerase complex [8,14]. Another example is the inactivation of the N-terminal 3'-exonuclease domain in two eukaryotic members of the polB family, pol α and pol ζ. Despite the overall high level of sequence conservation, the catalytic residues in the exonuclease domains of these polymerases are replaced with amino acid residues that cannot function in the proofreading reaction, so structural roles have been proposed for these domains [2,18]. To our knowledge, the PolB derivative described here is the first case of an evolutionarily conserved and, by implication, functionally important but, apparently, inactive DNA polymerase. Conceivably, this protein is involved in archaeal DNA replication, perhaps, as a structural subunit of the DNA polymerase holoenzyme [14,19]. Determination of the function of this unusual protein should yield significant insight into archaeal DNA replication.

## Competing interests

The authors declare that they have no competing interests.

## Authors' contributions

IBR and YIP made the original observation and performed initial sequence comparisons, KSM performed the final sequence analysis and phylogenetic analysis, EVK wrote the manuscript. All authors edited and approved the final version.

## Reviewers' comments

### Reviewer's report 1

#### Purificacion Lopez-Garcia, Universite Paris-Sud

Rogozin and co-workers report here an interesting observation. They identify a highly conserved clade of B-family polymerases grouping euryarchaeotal, crenarchaeotal and also some bacterial sequences whose Mg^2+^-binding motif has several replacements from the canonical YGDTD, notably the tyrosine and one of the aspartates that are essential during polymerization. They propose that these amino acid changes lead to inactivated enzymes that might have retained a purely structural role. This is a justified in silico prediction that, however, remains to be tested. In particular, it would be important to see whether the enzymatic activity actually disappears or whether some kind of activity can still be performed, or performed under particular conditions and with precise partners. One of the aspartates involved in polymerization remains very well conserved in this motif; the same is true for another aspartate upstream in the sequence. Such sequence conservation at long evolutionary distances might indicate more than an exclusive structural function. I hope that this observation will foster the appropriate biochemical work downstream. I would recommend the authors to insist on the hypothetical nature of their prediction, which they do in parts of the text by speaking about 'apparently inactivated' polymerases, through all of it including the title and the figure legend.

Authors' response: *We appreciate the comments and included additional qualifications in the text; we also emphasize the conservation and probable functional importance of the distal aspartate in the Mg^2+^-binding motif. However, we also stress the obvious disruption of the catalytic motifs in the exonuclease domain, an observation that suggests concordant inactivation of both domains of these proteins*.

### Reviewer's report 2

#### Chris Ponting, Oxford University

Rogozin et al. have made the interesting observation that some bacteria, euryarchaea and crenarchaea possess a homologue of B family DNA polymerase that contains substitutions within the YGDTD active site sequence. The evidence that this represents a homologue whose function is likely to be distinct from that of typical family B polymerases is compelling. What function this might be is uncertain, although the authors tentatively suggest an atypical role in DNA replication. This study shows, lest we ever forget, that demonstration of homology need not always lead to a successful prediction of function.

## Supplementary Material

Additional File 1 Multiple sequence alignment of active and inactivated forms of archaeal B-family DNA polymerases. Click here for file
