# Supplementary material for: A highly conserved family of inactivated archaeal B family DNA polymerases
Source: Biol Direct. 2008 Aug 6;3:32. doi: 10.1186/1745-6150-3-32 (PMC2527604; doi:10.1186/1745-6150-3-32)
Supplement: Additional File 1 — Multiple sequence alignment of active and inactivated forms of archaeal B-family DNA polymerases. [file 1745-6150-3-32-S1.doc]

**Supplementary Figure 1.** Alignment of the exonuclease and polymerase domains of archaeal B-family polymerases.

16081956 E Thermoplasma_acidophilum_DSM_1728 LKVLSFDVENEI--NR-----ENVEDYGKILVIGYSVSFQGKTVT--------------------------------GSLSGE---EQD--ILRSFVDLIRAE-DPDVITGYNIDG-YDI-PVIKKRMD-RY-----GIKLE-----IGRDGSI-------PRRIMNQF------WRVHGRLISDTWWSV----KRILHPK------HESLDYVANML---L

126007817 E Ferroplasma_acidarmanus_fer1 LKIFSFDIENSI--ST-----------REIFVIGYSVFF----------------NGEITEG----------------ALTGT---EPE--MLEDFNKLVQRE-DPDIITGYNIDG-YDM-PLLEERMK-FN-----KVRFS-----IGRDYIS-------PRRIMDQY------WRLHGRVIDDTWWEV----RKVLHPK------HETLNYVSNML---L

48477200 E Picrophilus_torridus_DSM_9790 LKILSFDVENSI--KT-----------KEIYVIGYAIYFNNK-----------------------------------IETGSITGTEHE--ILKKFNELIINN-DPDIITGYNIDG-YDL-PLIEERMK--YNNIKFGIGRD----------------HLPPKRILDQY------WRLHGRVVSDAWWAV----KKVVKPK------HETLNYVARLL---L

76801404# C Natronomonas_pharaonis_DSM_2160 SRLHILDIEVDD-RHG---FPEDGEE--EIVCLTSYDSYRDEYVIWLSESDDGVGGPEALGGYDPIGDGPLDVD---VRRFDE---EAT--MLVDYLDYIEDT-DPDVLSGWNFDD-FDA-PYLIDRID-RIASRHDRLYSD-------RLSRV-----REVWDSGWGG------PNIKGRVVFDLLYAYQ--RTQFSELD------SYRLDAVGEEE---L

15789745 E Halobacterium_sp-_NRC-1 LRVNTFDIEVND-RNG---FPEDGEE--PVVCLTSHDSYRDEYVAWLYAAPDATVDAPTAVPGYDPLTDDADID---VRVFDT---EEA--MHDAFLSYIEDT-NPDVLTGWNFDD-FDA-PYLIDRLDELDPRTDHDLDSD-------RLSRV-----SEVWTSGWGG------PNVKGRVVFDLLYAYQ--RTKYSELD------SYRLDAVGEQE---L

88602445 E Methanospirillum_hungatei_JF-1 TRYCLLDIECED--DK-GGLPNPDRD--RIICITAWDSFSDHYTTFLLQNSER-SINEENLCRNTLHNGCFRGDIHTIRVFDD---EKS--LLTGFSSYIREN-NPDILSGWNFTD-FDL-PFIFGRIR-AL-----NLPPD----------------SMARLPGMSER------SGVRGRVDFDLLAGYK--KMQSSKLD------SYRLDAVGERE--VG

154150651 C Candidatus_Methanoregula_boonei_6A8 ARVCMIDIECED-ERG---FPDAQRD--AIICITCYDSFDNDYTTFLLGSAILPGEIAEKEAAGGLKNGCFKKGVHTICTYDN---EAS--MLREFGAYIARR-DPDVLSGWNFVD-FDM-PYITGRME-KL-----GLSPV----------------LLARLPGMTER------NAVRGRALFDLLTGYK--KMHSTLKE------SYRLDAVAEEE---V

126178748 E Methanoculleus_marisnigri_JR1 ARTCIMDIECVD-EQG---FPEPERD--PIICVTCWDSFDDDYTTLLWQPGEAAGDAPDLCVQERHR----------VVRYPD---EIA--MLKGLVDYVKKR-DPDILSGWNFVE-FDI-PYIVKRMG-AL-----GLKAE----------------DLARIPGQTER------NAVRGRSIFDLLGAYR--KMHQAQKE------SYRLDAIAGEE---L

124485441 E Methanocorpusculum_labreanum_Z PRVCMCDIECDD-RNG---FPEPERD--PIICITCHDSFDDQYTTFVLNGKTKGGYGSAEPLASGCFSKCHT-----IISYDT---ERD--LFAGFIDYIKEK-DPDILSGWNFID-FDA-EYILKRAE-TL-----GFRSE----------------VFARMPGMTER------NAMRGRVIFDLLAAYK--KMQGSQKE------SYRLDAVAEDE---L

116754694 E Methanosaeta_thermophila_PT LRFMSFDIECLPQNGE---MPRPESS--PVILISMAFHPPYHGMSDLVLVGRELECDRPD-----------------VEGCAD---ERA--LISRFVSVIDDY-DPDIIAGYNSNE-FDI-PYLRERAS-RL-----GIEMN-----VGRDGST----WLIRSMGSNKN------VAVTGRVVVDLLPII----RSSFSLK------QYTLRNAAMEL---I

91773640 E Methanococcoides_burtonii_DSM_6242 LKYMAFDIECLPHDGG---MPTPDVS--PVIMVSFAFEPAYKGHKTLVLVMKEVEGVDED-----------------VEMLPD---ESD--LLNRFFEIFRDY-DPDVITGYNIND-FDV-PYITDRVK-LLNESGSNINSA-----VGRDSRA----LSYRKVVTRTM------VPIPGRVVVDSLPLI----RQQFSLK------RYTLRNVSKEL---L

21228106 E Methanosarcina_mazei_Go1 LKYLSFDIECLPLDGG---MPSPDVS--PIIMISFSFEPAYKGHKTLILLAKPADGMNGD-----------------VLPCKD---ETD--MLNRFFEIICEY-DPDIVAGYNHQD-FDI-PYITDRVK-ALNAKGENINPV-----VGRDGSH----IGYRRFGLITR------TEVKGRVVVDALPLV----RRAFSLK------QYTLRAVSKEL---L

41614864 N Nanoarchaeum_equitans_Kin4-M PSVLAFDIEVYS-----EAFPNPEKD--KIISIALYGDNYEGVISYKGEP---------------------------TIKVNT---EYE--LIEKFVEIIESL-KPDIIVTYNGDN-FDI-DFLVKRAS-LY-----NIRLP--IKLVNKKEPT---------------------YNFRESAHVDLYKTITTIYKTQLSTQ------TYSLNEVAKEI---L

84490287 E Methanosphaera_stadtmanae_DSM_3091 NKILSFDIEVYN-AEG---MPSAKKD--AIIMMSLCGNNGFKKVLSTKKSSKDF-----------------------VETLPT---EED--MLKRFVEIIKEE-NPDMLVGYNSDN-FDL-PYIKERADTLNIKLPLGIDKS---------------SIKFIKRGFNNA------GLIRGRVHVDLYLLV----RQYMNLD------RYTLERVYKEL---F

15679219 E Methanothermobacter_thermautotrophicus LDILSFDIEVRN-PHG---MPDPEKD--EIVMIGVAGNMGYESVISTAGDHLDF-----------------------VEVVED---ERE--LLERFAEIVIDK-KPDILVGYNSDN-FDF-PYITRRAA-IL-----GAELD-----LGWDGSK----IRTMRRGFANA------TAIKGTVHVDLYPVM----RRYMNLD------RYTLERVYQEL---F

15669075# E Methanocaldococcus_jannaschii_DSM_2661 LKSVAFDMEVYN-RDT---EPNPERD--PILMASFWDENGGKVITYKEFNHPN------------------------IEVVKN---EKE--LIKKIIETLKEY---DVIYTYNGDN-FDF-PYLKARAK-IY-----GIDIN-----LGKDGEE----LKIKRGGMEYR------SYIPGRVHIDLYPIS----RRLLKLT------KYTLEDVVYNL---F

45357943 E Methanococcus_maripaludis_S2 FKTVSFDIEVYC-NKE----PNPKKD--PIIMASFSSKDFNTVVSTKKFDHEK------------------------LEYVKD---EKE--LIKRIIEILKEY---DIIYTYNGDN-FDF-PYLKKRAE-SF-----GLELK-----LGKNDEK----IKITKGGMNSK------SYIPGRVHIDLYPIA----RRLLNLT------KYRLENVTEAL---F

57639936# E Thermococcus_kodakarensis_KOD1 LKMLAFDIETLY-HEG----EEFAEG--PILMISYADEEGARVITWKNVDLPY------------------------VDVVST---ERE--MIKRFLRVVKEK-DPDVLITYNGDN-FDF-AYLKKRCE-KL-----GINFA-----LGRDGSE----PKIQRMGDRFA------VEVKGRIHFDLYPVI----RRTINLP------TYTLEAVYEAV---F

6015025 E Thermococcus_gorgonarius LKMLAFDIETLY-HEG----EEFAEG--PILMISYADEEGARVITWKNIDLPY------------------------VDVVST---EKE--MIKRFLKVVKEK-DPDVLITYNGDN-FDF-AYLKKRSE-KL-----GVKFI-----LGREGSE----PKIQRMGDRFA------VEVKGRIHFDLYPVI----RRTINLP------TYTLEAVYEAI---F

14521919 E Pyrococcus_abyssi_GE5 LTFLAVDIETLY-HEG----EEFGKG--PIIMISYADEEGAKVITWKSIDLPY------------------------VEVVSS---ERE--MIKRLVKVIREK-DPDVIITYNGDN-FDF-PYLLKRAE-KL-----GIKLP-----LGRDNSE----PKMQRMGDSLA------VEIKGRIHFDLFPVI----RRTINLP------TYTLEAVYEAI---F

11498108 E Archaeoglobus_fulgidus_DSM_4304 LKMLVFDCEMLS-SFG---MPEPEKD--PIIVISVKTNDDDE-----------------------------------IILTGD---ERK--IISDFVKLIKSY-DPDIIVGYNQDA-FDW-PYLRKRAE-RW-----NIPLD-----VGRDGSN------VVFRGGR--------PKITGRLNVDLYDIA----MRISDIK------IKKLENVAEFLG--T

20094475 E Methanopyrus_kandleri_AV19 LVVASFDLEVLA-EPG--TTIKGASG--PIIAISFAYSTPDGERRNYVITWKGEDESFEVDGVETE-----------VIVCRS---EAA--ALRRFFDEFRRV-DPDVVFTYNGDE-FDL-PYLQHRAG-KL-----GIDVSPLARPAGKRGII------LKHGGGRYA------SDIFGRAHVDLYHTA----RKNLKLE------RFTLEEAVKDV---L

15897046 C Sulfolobus_solfataricus_P2 LRTIGVDFQIYS-KYG---SLNPRKD--PIVVMSLWSKEGPMQFSL-------------------------------DEGID----DLK--IIRRFVDYILNY-DPDIIFVYDSDL-LPW-KYITERAS-SL-----GVKID-----IGRKIGS------EVSVGTYGH------YSISGRLNVDLTGLL----VNERSLG------HVDLIDVSNYLG--I

146304901 C Metallosphaera_sedula_DSM_5348 LRFMAFDIEVFN-KYG---FPNPRRD--PIIVIGVWTDNGSKQF---------------------------------VNNDQD---DLK--ILRDFSKFVVEY-DPDVILGYNSNG-FDW-QYMLERAS-VR-----GTKLD-----IGRKVNS------EPSQGTYGH------YSVVGRLNVDLYGFA----ESLEGVK------VKSLDNVADYLGILP

159041906 C Caldivirga_maquilingensis_IC-167 LNYMALDIEVYN-PRG---TPDPKRD--PIIIIALANSNGDVKLLT-------------------------------LDNYKH---ERE--MLNDMMSVIKEW-DPDVLFGYNSNK-FDM-PYLVNRAD-AL-----NVKLQ-----LSKYGTP-------PEQSVYGH------WSIIGRAHIDLYNFI----EDMTDVK------RKSLDYVAEYFGVMK

119719310 C Thermofilum_pendens_Hrk_5 LKTYAFDIECYN-RYG---EPDPERD--PVLVISRTGEDGTV-----------------------------------IFSQDEGSSEKK--LLEEFVNDLVQY-DPDVILGYNSNR-FDW-PYLLQRAR-VN-----GVKLS-----IGRNLGE-------PSQSVYGH------FSVVGRANVDLYDYA----SELQEVK------VKTLENVSEFLGVMK

126465787 C Staphylothermus_marinus_F1 LRIIAFDIEVYN-KSG---TPRPQTD--PIIIIGIMNNNGDIKQFL-------------------------------ANKYD----DKI--SVEEFVNYVKTF-DPDIIVGYNTDG-FDW-PYLIERSK-YI-----GVKLD-----VTRRVGA------TPRTSTYGH------ISVPGRLNTDLYHFA----EEIPEVK------VKSLENVAEYLGVMK

124027770 C Hyperthermus_butylicus_DSM_5456 LRLTAFDIEVYN-KSG---SPNPNRD--PVIIIAVKSSNGDEKLFE-------------------------------ADGYD----DRK--AIRGFVDYIKSF-DPDIIVGYNSNG-FDW-PYLMQRAR-KV-----GVRLD-----VTRRVGA------EPTTSVYGH------VSVPGRLNVDLYHYA----EEIHEVK------VKTLEEVAEYLGVMK

156936857 C Ignicoccus_hospitalis_KIN4-I LKTLAFDIEVYN-KSG---TPRPERD--PIISIALASEGSV------------------------------------VSKLSQDKNDKD--LIVWFKKEMLER-DPDVVVGYNSNS-FDW-PYLIERSK-VV-----GVRLD-----VGRKVGV------IPTTGAYGH------VSVPGRLNVDVYDFA----KEVYEVK------VKTLENVADYFNVMK

118431730 C Aeropyrum_pernix_K1 LRVMAFDIEVYS-KMR---TPDPKKD--PVIMIGLQQAGGEIEILE-------------------------------AEDRS----DKK--VIAGFVERVKSI-DPDVIVGYNQNR-FDW-PYLVERAR-VL-----GVKLA-----VGRRSVE-------PQPGLYGH------YSVSGRLNVDLLDFA----EELHEVK------VKTLEEVADYLGVVK

18313103 C Pyrobaculum_aerophilum_str-_IM2 LRVLAFDIEVYN-ERG---TPDPLRD--PVILLAVQASDGRVEVFE-------------------------------ASGRD----DRS--VLRSFIDFVREF-DPDVIVGYNSNQ-FDW-PYLAERAR-AL-----GIPLK-----VDRVGGA-------PQQSVYGH------WSVTGRANVDLYNIV----DEFPEIK------LKTLDRVAEYFGVMK

170290810 K Korarchaeum_cryptofilum_OPF8 LKKLFFSGMIYS-EDG---FPVEGKS--PIVAISYSVDDGPIEVIS-------------------------------ASDLD----DSV--IIREFVSVISKE-NPDVIVGYGQDA-DEF-KHLMARSK-NL-----GLRLS-----IGRDGSEPMETGKFFRGTIIVE------NRIRGRANLDLFSVAW---RDFPQLP------ERTWYELADELG--V

**E-exonuclase; P-polymerase; N-nucleotide binding ......EEE......................................................................................................E......E.......................................................................................................**

170290793 K Korarchaeum_cryptofilum_OPF8 MPNAALDIEVLS--PL-DKIPDPREPDKPVAAAAIVDSSGRKEVHVLHRGGSLPGELSNGAK---------------LISYES---EGR--LIRSVLDRISEY---PLLFTYNGDN-FDL-PYLARRAK-ELGVSDRPVRLE-------------------KDRAILDT-----------GIHIDLYKLFSNRSIQVYVFDNKYA--GYTLDEVADAI---L

14600448 C Aeropyrum_pernix_K1 LPLIAFDIEVYS--PIATRLPDPSTAPYPVISAATADSSGRSRVVLLYRDGVEFTEGALPEGTE-------------VEIYDS---ERA--MLLDLVRILQRY---PLVVSFNGDN-FDL-PYIARRLE-VL-----GVPRE-----------------FAPIELKQDY------ATFRRSLHIDLHKLFGIRALQVYAFGNKYR--ELSLESISRAL---L

146304655 C Metallosphaera_sedula_DSM_5348 VKRVAIDIEVYT--PMMGRVPDPVKAEYPVISVALAGSDGLKLVLVLDRGDSPIQSKDIK-----------------VEVFRT---ERE--LLSRLFDILKEY---PMVLTFNGDD-FDI-PYLIFRGF-KLGLLQDEIPFE------------------ISSFGRKPD------AKFRYGFHIDLYRFFFNKAVRNYAFEGKYS--EYNLDTVAQAL---L

15897474 C Sulfolobus_solfataricus_P2 (1S5J 190-686) IKRVAIDIEVYT--PVKGRIPDSQKAEFPIISIALAGSDGLKKVLVLNRNDVNEGSVKLDGIS--------------VERFNT---EYE--LLGRFFDILLEY---PIVLTFNGDD-FDL-PYIYFRAL-KLGYFPEEIPID---------------------VAGKDE------AKYLAGLHIDLYKFFFNKAVRNYAFEGKYN--EYNLDAVAKAL---L

**Secondary structure (1S5J) E-β-strand; H-α-helix EEEEEEEE EEEEEEEE EEEEEE EEE EE EEEE HHH HHHHHHHHH EEEE HH HHHHHHHH EE EE EEEEHHHHH HHHHHH HHHHHHHH H**

126465771 C Staphylothermus_marinus_F1 AKRIAIDIEVYT--PFKGRVPNPSLAEYPIISIALVGTDNYRRVLVLARETMWGELSEEYPHNAE------------IEIFDS---EEA--LILEAIRVMTKY---PIVVTFNGDN-FDL-PYIFHRAL-RI-----GIPKE-----------------YLPIHFGEDI------VRLETSIHIDLYKFFKNRAIKSYAFGGKYQ--EENLDAIATAL---L

156937483 C Ignicoccus_hospitalis_KIN4-I VKAIAIDIEVFT--PAIGRIPDPETAQYPVISVALSSNDGLKKVLVLIRENLKLTEDQLKELAEEDYE---------VEFYDS---EKS--MLIEVMRIIRDY---PVLLTYNGDN-FDL-AYLYNRAL-KL-----GIPKE-----------------MIIFKKGSDK------FEIKHGIHIDLYRFYDIAAIKTYAFGNKYK--EVNLDAVAGAL---L

124028129 C Hyperthermus_butylicus_DSM_5456 PRRLAVDIEVYT--PFKGRVPDASAASYPIISVAFASDDGLRVVFILARPGVVGGEPREGVKEGVH-----------IEIFDD---ERA--LILETFRLIANY---PIVLTFNGDN-FDF-PYLYNRAL-KL-----GIPKD-----------------VIPFKPTRSY------MSLKYGIHIDLYQFFSIKAIQSYAFGNAYK--EFTLDAIASAL---L

159040732 C Caldivirga_maquilingensis_IC-167 LKRIAIDIEVYT--PQENKIPNPKEALYEIIAIGLAGSDGLKRVLVLRRPEILMNADDQGMLLSDDVE---------VMFFDN---ERD--LLREFFKTIMAY---PILITFNGDS-FDL-TYIYNRAL-KLGFRKEELPII---------------------LTRDGE------ARYVLGVHIDLYKFFNIRAVEVYAFGGKYSGGEKTLDAIASAL---L

171185814 C Thermoproteus_neutrophilus_V24Sta IRRVAIDVEIYT--PQENRIPDPKEAEYEVISVALVGSDGLRRVLMLRRPDV-----EAELRYRPDYE---------IMFFDS---EYD--LLREVFKTIVQY---PIVVTFNGDN-FDL-PYLYNRAV-ALGIPKEEVPIA----------------------VKRDY------VSVAPGVHIDMFKFFAIKAVEAYAFGGVYRG-ERGLDGIAYAI---L

18313158 C Pyrobaculum_aerophilum_str-_IM2 LKRVAIDLEVYT--PQENKIPDPKNAEFEIISVALVGSDGLRRVLLLKRPGLEIDLRYRPDYE--------------VLFFDS---EYD--LIREVFKIIVQY---PIVITFNGDN-FDL-PYLYNRAL-ALGMSKEELPIA----------------------AKRDY------VSVSPGVHIDTFKFFAIKAIEAYAFGGVYRG-ERGLDGIAYAI---L

119719216 C Thermofilum_pendens_Hrk_5 LRRVAIDIEVDS--PP-GIVPNPEKADRAVIAVSYYSDDRKGVLLLKRSNDIPKLEQNDAE----------------ITVFEN---EKE--LLQAAFKIMTSY---PVVLTFNGDN-FDL-PYLLNRAL-KLGLREEEIPIE--------------------WSPRSDY------AEIKGSIHIDLYKFFTNKSMRVYAFGNKYRE-GRTLDEVSMAL---L

161528456 C Nitrosopumilus_maritimus_SCM1 IKRLSVDIEVEA---EIGRIPDPKIAEKKVTAIGLKGSDGFDQIFVLKTEGTEQGTNELDQNIK-------------VTFYDL---DKEKEMIYDAFKIIKEF---PFVVTYNGDE-FDL-PYLYNRAE-RL-----GIKSS-----------------ENPFYMMRDS------ATLKHGVHLDLYRTFSNRSFQIYAFSQKYT--NFSLNEVSKAL---L

3599383 C Cenarchaeum_symbiosum IRRLSFDIEVDS---EEGRIPDAKISDRRVTAVGFAATDGLRKVLVLKSGADEGANDVTPGVE--------------VVFYDE---DKEADMIRDALAIIGSY---PFVLTYNGDD-FDM-PYMYNRAR-RLGVADSDIPLY----------------------MMRDS------ATLRHGVHLDLYRTFSNRSFQLYAFAAKYT--DYSLNSVSKAM---L

188997124 B Sulfurihydrogenibium_sp LRIMYIDFELGI-GKE--NLRFLRHI--PTLTIRFNEDEKSI-----------------------------------VIDN----------DLEYLQELLIKK-DPDIIIANYGDD-ILI-PNLFEKGL-KF---------------LNRNNLP----------------VIKKEGKSFV------------------------------------------

183221542 B Leptospira_biflexa_serovar- LRAISLYLE--K-SHR---HPF--QE--NTLIVETPKEKYNI-----------------------------------PTKN-----GIT--LIEKLNLILQKH-NPDIILSSFGDQ-VIF-PYLFKTAQ-EC-----HLTTE-----FDRDKTS------------LIRRSIQTQGTSFN------------------------------------------

124515762 B Leptospirillum_sp-_Group_II_UBA LRIVTLFPE--E-ERG---HPRHGLP--PALAIGTSDGIRVL-----------------------------------NGEE-----PVL--LLETLNNRIRQD-DPDLLLTSWGDD-WIF-PGLYALSA-RT-----GIPLE-----LSRT---------------AVTKKSSRKARTWF------------------------------------------

16120259 E Halobacterium_sp-_NRC-1 LSVPVTETS------N---DIY---E--ELSVAGDA-----------------------------------------VTGSST---D----ILAAVQGALEAH-DPDVLVCSTSEI---V-PTLHEMAT-DAGVDDFSLSRW-----------------------PDVDYQQLASRSTYS------------------------------------------

11500019 E Archaeoglobus_fulgidus_DSM_4304 FEVLRVGVK------G---DVF---K--PEVEEMSINGKR-------------------------------------IEGS-----EKE--LLEELSGAMEDM-NPDIVVMEDADF-WMQ-HLGLKAMS-RT-----GRFRK-------------------------------LSQKVYW------------------------------------------

88602132 E Methanospirillum_hungatei_JF-1 DQKADLDLMEITCRENPHRNPHPEHI--TIITSARQ-----------------------------------------WILDGP---ERT--IAEDLFTIIQDK-DPDIILFPDYDT-WSGIFFGIAREN--------YLFNA----------------------FSRTGRFHTVAPRSYF------------------------------------------

154150796 E Methanoregula_boonei_6A8 SPDVAHDLTILELRTR----GNPVVSA-PSLEFDLE-----------------------------------------CSGETCRLDGKAGDVLADLAGQIASH-DPDIILMPDADL--AV-PGLLTLAR-QY-----GITVP----------------------FSRNGKYRNQSARSYW------------------------------------------

16081572 E Thermoplasma_acidophilum_DSM_1728 --LSFFEMRSVYD-------PD------PDLNVAMVDCSHGKCIVD-------------------------------GEAMG----DGR--IYDRLMD--------SHVIVYEGTE-----QFYRKLMA-------LGIRAR------------------------------YYPGKSFT------------------------------------------

126008334 E Ferroplasma_acidarmanus_fer1 ----EFYSLK---------NPYEEDISLPAAVIEPVSRYGRIKYVA-------------------------------INGNRY---SSN--IYEETKRAI----EENIIIIYRNYQ-NEF-SILLDQMK-KH-----GYYIN----------------------------ARFYRERTFE------------------------------------------

126465179 C Staphylothermus_marinus_F1 LKKRGEGYVALEN------PFDPEYSEPPIKYLILRLFRDGYRLYSETIDPEKAVIENSE-----------------GIVYE---------------------GDLDGAVEVVREYKPLILY------------------------------------------------TSIVEKIYVEQ-----------------------------------------

118431787 C Aeropyrum_pernix_K1 LDDYGVLSLAVVEAY----SWHGPISSPLEKPG---RFRVVC-----------------------------------GGWEEAV-------------------GGVDGALDVLRGCRPHVVV-------------------------------------------------ARSPVRLILE-----------------------------------------

146304209 C Metallosphaera_sedula_DSM_5348 KVLEFWDLGLSF----------PPVKYATVITHDWYGPSET------------------------------------GNMFEA---------DVNGEKWRGLLRDLDLEVDVAGCFGRACDH--------------------------------------------------------VK------------------------------------------

15898291 C Sulfolobus_solfataricus_P2 ----------EF----------PKVTIARVVPLDWYGESLK------------------------------------GKVFEV----------KINNEVRRFYEKPEVEVDIAECLGEACNY--------------------------------------------------------VK------------------------------------------

16081956 E Thermoplasma_acidophilum_DSM_1728 GEGKDNIDRLHIEDEWKKRR---EEVIAYCIKDADLTLRI--FEKLMVMNRLMYMSSVTKLPLDDVANVGTSNYVDSILIRAADRENIGVPMNQH-------------EIKTEEIQ--GGYVHSI-G-AGL-YSNVIVLDFKSMYPSMIIKYNVCFTTLD-----------------PNGEIL-SPN-GVRFLSPEKKKGLIPRILQELMADRDEVKRRMKA

126007817 E Ferroplasma_acidarmanus_fer1 NEGKEDVNRLDIENEWKNRR---DDVIKYCIKDARLALLI--YRKIRILDRNLYLSTVSMLPLDDVTNGGTSTYVDSLLIRRADRENIGVPMSSS-------------KFSNDTYA--GGYVHTM-D-PGL-YDMIVVLDFKSMYPSMIIKYNICFTTLS-----------------PEGTIV-APN-GARFLDPGVKKGIIPDLLEQLMKNRDEVKKQMKE

48477200 E Picrophilus_torridus_DSM_9790 NDEKENIDRINIESEWEAKK---EDVIKYCIKDAYLALEI--YRKIRILDMNLYLSAVSKLPLDDVANGGTSTYVDSILIREADKQNIAVPMSAH-------------DFKEESFE--GGYVHSI-G-AGL-YDMVIVLDFKSMYPSMIIKYNICFTTLN-----------------RNGTIV-APN-GARFLSPDIKKGLIPSMLESLMKDRDNIKKQMKS

76801404# C Natronomonas_pharaonis_DSM_2160 GVGKERYPG-DIGDLWEDDP---ERLLEYNLRDVELCVEL--NRKQNIVEFWEEVASFVGCKLED--ATTPGDAVDMYVLHKIHG-EFALPSKG--------------HQEGEEYE--GGAVFDP-I-SGV-KEMVSVLDLKSLYPMCMVTINASPETKV-------------DPDNYGAETYAAPN-GTHFRKEP--DGVIREMVDELLEEREEKKSLRNE

15789745 E Halobacterium_sp-_NRC-1 GVGKERYPG-DIGDLWEDDP---ERLLEYNLRDVELCVEI--DRKQSIVAFWDEARKLVGCKLED--ATTPGDAVDMYVLHKAFG-NFVLPSKG--------------QQDAEEFE--GGAVFDP-I-TGV-RENVSVLDLKSLYPMSMVTINASPETKV-------------DPEEFDGDTYRTPT-GVHFRKEP--DGIIREMVDELLTEREEKKARRDD

88602445 E Methanospirillum_hungatei_JF-1 DVKAFHYQPGMTARMWNEAP---HELVEYNFKDVELCVKI--NQKNNIIEFYQEIARYVGCPLDR--TLNSSNVIDIYILRKAHG-KFILPSKG--------------YSPGDEFE--GATVFEP-S-SGI-RENVVVLDLKSLYPMAMMTINASPETKD-----------------PNGDLI-APN-GIKFRSKP--DGLTRSIISELLQERDLKKNKRNE

154150651 C Candidatus_Methanoregula_boonei_6A8 GERKVRYTG-TISDLWRKQP---ALLVEYNFKDVELCVAI--DRKDSIVSFYREIARYVGCPLDK--TLNSSSVIDVYILRKAHG-KYILPSKG--------------FANAEEFE--GATVFEP-S-KGV-RENVVVLDLKSLYPMAMMTINASPETKD-----------------PEGELK-APN-GIRFRKQP--DGLTRSILSELLKERDAKKNLRNT

126178748 E Methanoculleus_marisnigri_JR1 GVTKVRYTG-TITDLWRTDP---KRLVEYNYRDVELCVGI--DQKNNIIEFYREIARYVGCPLDR--TLNSSNVIDIFVLRKASG-TFVLPSKG--------------LAAGDEFE--GATVFEP-A-TGL-RENVVVLDLKSLYPMAMMTINASPETKN-----------------PDGELR-APN-GIRFSREP--DGLTRSIIAELLEERDERKRLRNL

124485441 E Methanocorpusculum_labreanum_Z GETKVRYIG-TLGDLWDNDP---LKMVEYNFKDVELCVGI--NRKNNIIEFYQEVARYVGCPLDK--TLNSSNVIDIYILRKAFG-KFILPSKG--------------NASGEEFE--GATVFDP-S-KGV-RENVIVLDLKSLYPMAMMTLNASPETKS-----------------PDGEIH-APN-GIRFRKSP--DGLTRSIISELMAERDERKKLRNS

116754694 E Methanosaeta_thermophila_PT GEEKRDMDPARMESIWLGGDGL-ADLIRYSRRDAVLVMQL--LLRLRLMDKYIALARVSGSLLQDIVNGGQSGMVENLILRRFRSHKRVLPPKPDSEESGE--------RFTDADELKGGAVLPP-V-KGL-VENVVILDYKSLYPTIMMAHNLCYSTVV--------------TKERPPEVVKSPS-GGYFASPSVCKGIVPEILRELLEKRTETKMLMKS

91773640 E Methanococcoides_burtonii_DSM_6242 GREKLDVDAADMEEHWNDSGEKIRKFIDYSRRDSELALEL--LLDLKLLDKYIALSRVSGILLQDTVSGGQTNMVEHILLTEFGKQGRVMSTKPDEETSS--------FRRKQNEDLKGGAVLEP-E-KGL-HKNVIVLDYKSLYPTIMMAHNLCYTTVV-------------PPGKYPEDKVIRSPSKGEFVKPEVFKGIVPSVLESLLDKRIETKKLMKQ

21228106 E Methanosarcina_mazei_Go1 SREKLDVPPLEMEEHWNDSGEKFLKFVDYARRDAELALEL--VLNLRLLDKYIALAQVSGSLLQEIVDGGQTSMVETLLLREFGLRDRVILPKPGDELSA--------ERYDMSSDLKGGEVLEP-K-KGL-LENVLILDYKSLYPTIMMAHNLCYTTVV-------------TKDRPDGEAIKPPS-GGEFVPPEVYRGIVPSILEDLLNQRGETKKRMRL

41614864 N Nanoarchaeum_equitans_Kin4-M GEEKI-YDYENMLYDWAIGNY--NKVFEYNLKDAELTYKL----FKYYENDLLELARLVNQPLFDVSRFSYSNIVEWYLIKKSRKYNEIVPNKPKMEEVER--------RKLNTYA--GAFVYEP-K-PGL-YENLAVLDFASLYPSIILEHNVSPGTIYCEHDDC--------KQNGVEAIINNEKKYVWFCKKV--KGFIPTVLEHLYTKRLELKRKLKE

84490287 E Methanosphaera_stadtmanae_DSM_3091 DIEKIDVPGDKIFEYWDSDNELLEKLFDYSMDDAITTTEI----ADKLTPLTVAQTRLVGQPLFDIARMTTGQMVEWYLIWKAFEKNNIIPNKPTTNEYTQ-------RRSSKKVA--GGYVKEP-E-KGL-FEHIAYLDFKSLYPSVIIAQNISPDTIT-----------TDDTLDESEYHLCPES-DYKFLKEP--KGFIPSIIGYILDERQRIKKLMYE

15679219 E Methanothermobacter_thermautotrophicus GEEKIDLPGDRLWEYWDRDELR-DELFRYSLDDVVATHRI----AEKILPLNLELTRLVGQPLFDISRMATGQQAEWFLVRKAYQYGELVPNKPSQSDFSS--------RRGRRAV--GGYVKEP-E-KGL-HENIVQFDFRSLYPSIIISKNISPDTLT--------------DDEESECYVAPEY-GYRFRKSP--RGFVPSVIGEILSERVRIKEEMKG

15669075# E Methanocaldococcus_jannaschii_DSM_2661 GIEKLKIPHTKIVDYWANND---KTLIEYSLQDAKYTYKI----GKYFFPLEVMFSRIVNQTPFEITRMSSGQMVEYLLMKRAFKENMIVPNKPDEEEYRR--------RVLTTYE--GGYVKEP-E-KGM-FEDIISMDFRSLYPSIIISYNISPDTLD--CECC--------------KDVSEKILGHWFCKKK--EGLIPKTLRNLIERRINIKRRMKK

45357943 E Methanococcus_maripaludis_S2 DVKKVDVGHENIPKMWDNLD---ETLVEYSHQDAYYTQRI----GEQFLPLEIMFSRVVNQSLYDINRMSSSQMVEYLLLKNSYKMGVIAPNRPSGKEYQK--------RIRSSYE--GGYVKEP-L-KGI-HEDIVSMDFLSLYPSIIMSHNLSPETID--CTCC----------SDEENGENEEILGHKFCKKS--IGIIPKTLMDLINRRKKVKKVLRE

57639936# E Thermococcus_kodakarensis_KOD1 GQPKEKVYAEEITTAWETGENL-ERVARYSMEDAKVTYEL----GKEFLPMEAQLSRLIGQSLWDVSRSSTGNLVEWFLLRKAYERNELAPNKPDEKEL---------ARRRQSYE--GGYVKEP-E-RGL-WENIVYLDFRSLYPSIIITHNVSPDTLN--REGC------------KEYDVAPQV-GHRFCKDF--PGFIPSLLGDLLEERQKIKKKMKA

6015025 E Thermococcus_gorgonarius GQPKEKVYAEEIAQAWETGEGL-ERVARYSMEDAKVTYEL----GKEFFPMEAQLSRLVGQSLWDVSRSSTGNLVEWFLLRKAYERNELAPNKPDEREL---------ARRRESYA--GGYVKEP-E-RGL-WENIVYLDFRSLYPSIIITHNVSPDTLN--REGC------------EEYDVAPQV-GHKFCKDF--PGFIPSLLGDLLEERQKVKKKMKA

14521919 E Pyrococcus_abyssi_GE5 GKSKEKVYAHEIAEAWETGKGL-ERVAKYSMEDAKVTFEL----GKEFFPMEAQLARLVGQPVWDVSRSSTGNLVEWFLLRKAYERNELAPNKPDEREYER--------RLRESYE--GGYVKEP-E-KGL-WEGIVSLDFRSLYPSIIITHNVSPDTLN--RENC------------KEYDVAPQV-GHRFCKDF--PGFIPSLLGNLLEERQKIKKRMKE

11498108 E Archaeoglobus_fulgidus_DSM_4304 KIEIADIEAKDIYRYWSRGEK--EKVLNYARQDAINTYLI----AKELLPMHYELSKMIRLPVDDVTRMGRGKQVDWLLLSEAKKIGEIAPNPP---------------EHAESYE--GAFVLEP-E-RGL-HENVACLDFASMYPSIMIAFNISPDTYG--CRDD--------------CYEAPEV-GHKFRKSP--DGFFKRILRMLIEKRRELKVELKN

20094475 E Methanopyrus_kandleri_AV19 GVEKEEMELADINEAWKRGNL--DELMRYSAEDAHYTLEL----GLELAQVELELSYLTRLPLPDATRFSFGQLAEWRAIYKARQEDILVPNKPTRDEYKR--------RRRKAYK--GAIVFEP-E-IGL-HENVVCVDFASLYPNVMVAHNISPDTFD--CDCC-PRVTVEEVDDPTDATVAPDV-GHKFCKRR--KGFFPRLVEGLIERRRELKRRLRK

15897046 C Sulfolobus_solfataricus_P2 SPSRYSFKWYEISRYWDNEKNR-RIIREYSIENARSIYLL----GNYLLSTYSELVKIVGLPLDKLSVASWGNRIETSLIRTATKSGELIPIRM--------------DNPNRPSKIKKNIIIQP-K-VGI-YTDVYVLDISSVYSLVIRKFNIAPDTLV--KEQC------------DDCYSSPIS-NYKFKREP--SGLYKTFLDELSNVRDSNKIKVIE

146304901 C Metallosphaera_sedula_DSM_5348 KNKRTNLEWYQIPEYWEDPKRR-EVVLKYNLDDVKTTYLL----RDVFFNFGEQLTVISGLPLDQLCMASVGHRVEWLLMRQAKQFNELIPNRV--------------ERRYEGYK--GGLVIEP-K-PGL-HENVAVLDFSSMYPSIMIKYNIGPDTLV--QGEC------------NDCWVAPEV-GYKFRKDV--DGFYRSILNFLLEERRKTKDQMSQ

159041906 C Caldivirga_maquilingensis_IC-167 RSERVNIPGHRIYQYWDDEGKR-SQLIKYARDDVLSTLGL----GKILLPYAMQLASVSGLPLDQVGPASVGSRVEMMIMHEAYKMGELAPNRV--------------ERPYETYK--GAIVLEP-K-PGI-HYNIAVLDFSSMYPNIMLKYNISPDTLV--LDSS------------EGDYYTAPEVGYRFRKSP--RGLYASLLQKLIEARREARDEMRN

119719310 C Thermofilum_pendens_Hrk_5 KSERVLIDTNKVYEYWDSKDKR-SLLLRYAGDDARSTYLL----GQVVLPFGIQLSSLVGLPLDQVFAASVGNRVEWFLIRQAFVFNELVPNSR--------------ERGEETYK--GAIVLKP-K-PGV-HKKIAVLDFSSMYPNIMIKYNISPDTYV--PPEE--------HVDPSEVWVAPEV-GHRFRKHP--PGFYRKVLESLLEARRRLREKMKT

126465787 C Staphylothermus_marinus_F1 KSERVIIEYIDIPKYWDDEKLR-PKLLQYNIDDVKSTYGL----AEKFLPFAMQLSNITGLPLDQVGAASVGFRLEWYLMREAFRYGELVPNRV--------------ERAAESYR--GAVVLKP-V-KGV-HENIAVLDFSSMYPNIMIKYNVGPDTIV-RNEKC----------NPDKHNIAPEV-GHCFRKEP--PGFFKRVLETLLRLRKQIKSEMKK

124027770 C Hyperthermus_butylicus_DSM_5456 KSERVLIHWWEIPRYWEDPEKR-PILEQYARDDVRATYGL----AEKMLPFAIQLSTVTGIPLDQVGVMGVGFRLEWYLIRAAYEMNELVPNRV--------------ERHEESYR--GAVVLKP-L-KGV-HENVVVLDFSSMYPNIMIKYNVGPDTIVDDPAEC---------EKYGGCYVAPEV-GHRFRKQP--PGFFKTVLENLLRLRKQVREKMKE

156936857 C Ignicoccus_hospitalis_KIN4-I KDERPLIPHHLIYQYWEDPQKR-DVLRKYNEADALSTLML----AELFIPFGEQLSYLTGLPLDQVMAASVGYRVEWYLMRVAFVTNELVPNRV--------------ERKVASYK--GAIVLRP-L-KGV-HENVAVLDFSSMYPNIMIKYNVGPDTLV--RPGE--------KVSPDEVYVSPA--GYMFKKRP--DAFFKRSLVTLLNLRKEIKERMKS

118431730 C Aeropyrum_pernix_K1 IGERVTLEWWQIGEYWDDPSKR-EILRKYLRDDVRSTMGL----AEKFLPFGAELSQVSGLPLDQVMAASVGFRLEWRLIREAAKLGELVPNRV--------------ERSEGRYA--GAIVLRP-K-PGV-HEDIAVLDFASMYPNIMVKYNVGPDTLV--RPGE--------EYGEEEVYTAPEV-GHKFRKSP--PGFFKKILERFLSWRRQIRSEMKK

18313103 C Pyrobaculum_aerophilum_str-_IM2 REERVLVPGHKIYEYWRDQGKR-PLLRQYVIDDVKSTYGL----AEKLLPFLIQLSSVSGLPLDQVAAASVGNRVEWMLLRYAYRLGEVAPNRE--------------EREYEPYK--GAIVLEP-R-PGL-YSDVLALDFSSMYPNIMMKYNLSPDTYL--ERGE--------PDPPGGVYVAPEV-GHRFRREP--PGFIPLVLRQLIELRKRVREELKK

170290810 K Korarchaeum_cryptofilum_OPF8 GRPEVMMKF-RVAEAWKLNR---DNALDYMRKKLSTIIGI----YSKIIDNQIALSKMSLIPIHKLLRSSVGEIVEAILLKESRSRDWIAFSSP--------------QRPEESYE--GGFVWIK-S-PGV-YEGICYLDFASMYPSIMALHNLSFETVN--PEEG----------FCEVEEVNVEGVRAKVCRDV--EGLVPQLVRKLIEERSKIKESLAN

**E-exonuclase; P-polymerase; N-nucleotide binding ................................E..........................................................................................................P..................................................................................**

170290793 K Korarchaeum_cryptofilum_OPF8 GEKKMELESRDFYSVET------RTLAEYCLKDAELTFRLGNIGSGEIIKLLFLFSRISKMSLEEVSRQGVSSWIRNMIFFEYRRRGWLIPEKEEIMKVRGERTYSQAIIKGKKYM--GAIVLEP-I-PGI-HFDVAVMDFASLYPSIIGRWKVSFETINCPHEDC------------RENRPVNEL-PHWICKKE--RGIVPYLIQALRDLRVKRYKRRAK

14600448 C Aeropyrum_pernix_K1 GKGKVELKAPVSELNL-------NKLIEYNLQDARLTLELLTFSNNLVFNLIIMVMRTSKLGIEDITRSQISNWIRGLMYWEHRRRRWLIPSRGEIEKLSSAGARVGAIIKDKKYR--GAIVLDP-P-VGI-FFRVLVLDFASLYPSLIKQWNLSYETVN--NPNC------------RDTIEVPEV-GHRVCREF--KGISNEIVGMLRDFRVRLYKKKSK

146304655 C Metallosphaera_sedula_DSM_5348 GLSKVKLDESISDLNM-------SKLVEYNYRDSEITLKLTTFNNELVWKLIVLFSRISKLGIEELTRTEISAWVKNLYYWEHRKRNWLIPLKEEILERSS-GLKTAAIIKGKGYK--GAVVIDP-P-VGV-YFDVVVLDFASLYPSIIRNWNLSYETVD--VKEC---------NKKRDIRDESGAKIHEVCVDR--PGITAVVTGLLRDFRVKIYKKKGK

15897474 C Sulfolobus_solfataricus_P2 (1S5J 190-686) GTSKVKVDTLISFLDV-------EKLIEYNFRDAEITLQLTTFNNDLTMKLIVLFSRISRLGIEELTRTEISTWVKNLYYWEHRKRNWLIPLKEEILAKSS-NIRTSALIKGKGYK--GAVVIDP-P-AGI-FFNITVLDFASLYPSIIRTWNLSYETVD--IQQC--------KKPYEVKDETGEV-LHIVCMDR--PGITAVITGLLRDFRVKIYKKKAK

**Secondary structure (1S5J) E-β-strand; H-α-helix H HHHHHHHHHHHHHHHH HHHHHHHHHHHHHHHHH HHHH HHHHHHHHHHHHHHHH HHHHHH EE EEEEEEEEE HHHHHHHH EE EE HHHHHHHHHHHHHHH HHHHH**

126465771 C Staphylothermus_marinus_F1 GISKIGVEGNIGELSL-------ANLVAYNYRDAEITLMLTLFNNELVWRLMILLARIAKVSIEDICRKSISKWIQNLFYWIHRKLNYLIPEPEDIRKYGK-PSTTQATIEGKKYA--GALVIEP-P-KGV-FFKVTVLDVASLYPSIIKKYNLSYETVD--KPNC--------KSKIDILDETGKK-IHHVCIDK--PGLSAQITGLIRDFRVGIYKKKAK

156937483 C Ignicoccus_hospitalis_KIN4-I GEHKVQLTKSISELNY-------YELAHYNFRDANLTLKLFTFNDYLPWKLMVLIARISKLGIEDLTRKQVSAWIKNLFFWEHRRRKYLIPNKEDIISMKG-TVKSSAIIKGKSYQ--GAFVFEP-S-AGI-FFNVVVLDFASLYPTIIKQYNISYETVN--APKC------------KNYYEVPEV-GHRICKDV--EGITSQIVGLLRDYRVKIYKKKAK

124028129 C Hyperthermus_butylicus_DSM_5456 GEHKVEIESTVSDLSL-------LELVRYNIRDAELTLKLTSFNRDLVWKLIVLLARISRLPIEDVTRSQVSAWIKSLLYWEHRRRGYLIPSRDEIIKLKG-EVKSEALIKGKKYQ--GAIVLDP-P-SGV-FFRVVVLDFASLYPSIIKKWNLSYETVN--PVYC----------PGSKIVEVPDV-GHKVCMSN--PGLTAQIVGLLRDYRVKIYKKKAK

159040732 C Caldivirga_maquilingensis_IC-167 GISKVEREKPISEMNY-------IELVNYNFRDALLTLYLTTFNNELVMRLIVLLARISKTPLNDVSRYQVSAWIRNMVYFEHRKRGWLIPNKEDVVNAKG-SVATKAIIKNKKYA--GAIVIEP-I-AGI-YPNVYVLDFASLYPSIIKRWNLSYETVRCPDEKQ---------ANDPRNRPVPNL-PHWVCSNM--RGLTSLLVGLLRDMRVYVYKKLAK

171185814 C Thermoproteus_neutrophilus_V24Sta GVGKVERQKNVSRMGY-------WELAEYNYRDALITLYFTLYNGEMVMKLIMLLSRIAKMPIEDITRSQVSAWIRNMLYYEHRRRGWLIPNKEDILREKG-SVHTKAIIKGKKYA--GAVVLDP-P-LGV-FFNVYVLDFASLYPSIISKWNLSYETVNCRQDAE---------------RPIPEL-PHTICRDR--PGLTSTLVGILRDLRVHVYKKLAK

18313158 C Pyrobaculum_aerophilum_str-_IM2 GIGKVERQKNISRMNY-------WELAEYNYRDALITLYFTMYNNEMVMKLILLLSRIAKMPIEDITRSQVSAWIRNMLYYEHRKRGWLIPNKEDILNVKG-QTHTKAIIKGKKYA--GAVVLDP-P-LGI-FFDVYVLDFASLYPSIISKWNLSYETVNCRPDAE---------------RPIPEL-PHTVCHDK--PGLTSTLVGILRDLRVHVYKKLAK

119719216 C Thermofilum_pendens_Hrk_5 GIGKVKHEEAISEMTP-------ERLVEYSLRDAELTYKLTSFNNDLVMKLIILMMRISKLPIEDLTRHNISAWIRNMLYYEHRQRGWLIPNLEDILRLKG-QTSTKAIIKGKKYL--GAIVINP-K-PGV-YFNVVVVDFASLYPSVIKTWNLSYETVRCQHKEC-------------MENRVPGT-NHWVCKKR--RGLMSEIIGILRDLRVYVYKKGAK

161528456 C Nitrosopumilus_maritimus_SCM1 GKEKIDYGLEFDQLTL-------YQTANYCYNDALLTYELTSFNSELLMQLLVIIARIGRMPIDDIARMGVSQWIRSLLYYEHRKRNCLIPKRQELERRSE-GVMSDAVIKDKKYR--GGLVVEP-K-EGI-HFDVVVMDFASLYPSIIKVRNLSYETVRCPHEDC-------------KRNEVPGT-NHWTCSKK--NGLTSMIIGSLRDLRVNYYKSLSK

3599383 C Cenarchaeum_symbiosum GEGKVDYGVSLGDLTL-------YQTANYCYHDARLTLELSTFGNEILMDLLVVTSRIARMPIDDMSRMGVSQWIRSLLYYEHRQRNALIPRRDELEKRSQ-QVSNDAVIKDKKFR--GGLVVEP-E-EGI-HFDVTVMDFASLYPSIIKVRNLSYETVRCVHPEC-------------RKNTIPDT-NHWVCTKN--NGLTSMIIGSLRDLRVNYYKSLSK

188997124 B Sulfurihydrogenibium_sp SYGQTVYTADM------------SLLLGRIHIDSRNSFFY----KEVGLDGIIELSRISSLGMQAVARTTIGTPITYMEMKNAYENNFLIPYKKNQPEDFKTL-----EQLIKIDK--GGLTLKP-L-VGL-FENVAEYDFFSMYPSIITNYNLSYETIN---CKH----------PECK-KTLPYT-NYRICTKK--IGIVPQTLKWLLERRLKYKQLLKK

183221542 B Leptospira_biflexa_serovar- TYGTIVFRAPS------------YPLFGRWHIDSRNSFVY----KEAELIGIIELSRISRLPIQKMARASTGKALTYIEVDVALRMNYLVPWQKSALESEKSA-----LQLLNADK--GGLVFQADIQNGFVLENVAQLDFSQMYPNIMVKHNISPETIN---CLC-------CEDDPNV-EKVPSL-GYRICAKR--KGIVSEALAHIVQRRNHYKEQ---

124515762 B Leptospirillum_sp-_Group_II_UBA SYGKVHFRPGA------------CLLSGRWHIDRTTSFIL----KEAGLDGLFEQARLTRIPVQDMARTSTGTGITSLQLALAVRKKILIPWRKNEPESFGTA-----LELLETDK--GGMVFLP-P-PGF-HESVAELDFASMYPSLMVRFNISPETVG---CSC----------CPGS--RAPGT-RHTICTRR--SGFIPEVLAPLLEKRQTYKDRLAN

16120259 E Halobacterium_sp-_NRC-1 SYGRVGHSPAR------------YNVPGRAIIDESNTFFY----GETNLDGVLDLVSRSKNPVQELAWASIGNVLTAIQICEANDRGVLVPWNSWRHEFYK-------PMGTLHDADRGGFIFAP-E-VGL-HENVHELDFSSLYPNIICTRNVSPDVIR---CDC-----------HSDRDDVPGL-GYSICDDR---GYLVDVLQPIIDARDEIKAAIRR

11500019 E Archaeoglobus_fulgidus_DSM_4304 SYGRARYRKQA------------LIPEGRVLIDS-NSFNF----REGGLRGVLLASRISAISPNHASRITPGSLISLYEVFEALMRGIAVPFRKSDAERVKSI-----EELRFSDR--GGMIFQP-E-PGI-YENVWQLDFTSMYPSIIVKHNLSPESIG---------------------------------KEG--RGFLAEVLEPLLRLRIKTKSLK--

88602132 E Methanospirillum_hungatei_JF-1 SYGRMEHRLGA------------KIPEGRIIIDTKQSFMY----REGDLRGIFLASRLAGLSPNLTCRLTPGTLISSYEVYEALARGYAVPFRKSDAEACR-------RVTAMRLDYRGGYILQP-P-PGI-FADVTQIDFTSFYPSIIVSENLSPETLN--------------------------------DRTK--PGFLSSVLEPVITLRQHTKQMKKT

154150796 E Methanoregula_boonei_6A8 SYGRMEHKGAA------------LVPEGRILIDTEQSFAY----HAGGLSGMLIASRLAGIPPGPASRLTPGTLISSYEIREALRQGIAVPFRKNEPEQVR-------KIAELQAADRGGLMFQP-V-PGT-YESVDEIDFTSLYPTIIVNENLSPETLG--------------------------------HNDR--PGFLPAVLDPVIGLRRTTKRQKKT

16081572 E Thermoplasma_acidophilum_DSM_1728 SYGQISYRDSY------------LDIADRIAINSR-SFFY----AESGLSGIYEVSRISHLPPLYVSIVTPGTAVSSMELAEAVKKGMLVPFKKDDHENPK-------TKWEIMAKDRGGLIFQP-L-PGL-YTDVYEIDFSSMYPSIIVRYDLTPGRIS----------------------------------------FLPEALEGLLSRRLIYKKMDGE

126008334 E Ferroplasma_acidarmanus_fer1 SYGRHSYMPGA------------VKIRDRICIDSD-SFVY----REAGIPGLFELSRVSSLPVEIVSSITPGTVVSSIEEKEALKRKILVPFRKDDYEIAK-------NPFELFSADAGGIVFNP-E-PGI-YSDVYEIDFSSMYPGIIVEYGISPESIS---------------------------------GGN--TAYLSQFLRGLLDRRLLYKYVNSR

126465179 C Staphylothermus_marinus_F1 RYSWIRKYYD-------------------VWIDDVE--------TLVDHTGIVYWSRLAYTPPRMLNYATIGRILTTIEALEARKHKYLIINGFGRRESWR-------SLRSLLETDRGGLVYSP-R-PGL-YWGVCQIDYNSLYPSIIAKYNISGETID---------------NPYCRKKHVVKNLPHIICLDR--RGLVSIVLNKLVKLREKAKMLVAQ

118431787 C Aeropyrum_pernix_K1 GSSRFREYLW-------------------FEPDR----------NLVGPRGLIVWMRISWLPYTMAHGAPIGRVLTAAEAREAYRRRYLLDERAPRHERFR-------SLRSLVESDMPGAARLP-E-PGV-YWGVVQLDYSSLYPRLIAMYNISAETVD---TPC-----------GNTLDPAPGYGGHRVCMDR--RGLVPEVLGRLVDLREEARRRGAG

146304209 C Metallosphaera_sedula_DSM_5348 APVKIKLEQKR---------------------------------SPVSIKGLIEWSYTCKTPVRELVDATIGKALTTNEAWVAFEKKIVVPNKVPRVEKLR-------DMDELLLNDKGGLVLFP-R-TGC-FDDAWQVDFSSMYPSLIVKHNISGETVD------------------ACDDVVTEI-GHTICNSE--RGIVPEALSWLIRRKEALKPVDPE

15898291 C Sulfolobus_solfataricus_P2 SNVKIRIEKKR---------------------------------SPVSAKGLIEWSFISLTPIHEIAYATIGKVLTINEAWVAFKRRIIIPKVVPRVEKLR-------RLEDIMMVDKGGLILFP-Q-PGC-YDNVYQVDFSSMYPSLIVKHNISAETVE------------------ACDDIKTEL--HSICLKE--KGIIPEALQWLIERKSELKKIDEE

16081956 E Thermoplasma_acidophilum_DSM_1728 -----AKTKDEREFYDGIQNAIKVLMNTFYGVLASSFYRFTDHKIGSAITAFARETIKGIISTLE-SAH-------------------------------------YRVIYGDTDSVFVES----------GAGSAEDAIKRGKDLSERLSREQ-------------G-LTLDFQMVLDPFFS--------HGAKKRYAG-------RCVYPD----------------DMKGEIVIKGYEVRRT

126007817 E Ferroplasma_acidarmanus_fer1 -------DKSNHDYLDGLQAAIKVLMNTFYGVLGTSFYRFTNREISSAITAYARNTITSIIADLD-KSG-------------------------------------NKVIYGDTDSIFIES----------GKKTLDEAIEYGNRLSSDISRKE-------------N-LVLEFEKIMDPLFS--------HGAKKRYAG-------KIVYPP----------------SSAGEILVRGYETRRT

48477200 E Picrophilus_torridus_DSM_9790 ------SSGEEKEYLNGLQNAIKVLMNTFYGVLGASFYRFTNPEIGGAITSLGRVTIKNIIDKLE-SSG-------------------------------------HRVIYGDTDSIFIES----------GKKTKEDAIKFGNELSDRISKEEN--------------LVLEFEKIMDPLFS--------HGAKKRYAG-------KVIYPD----------------SMAGEILVRGYETRRT

76801404# C Natronomonas_pharaonis_DSM_2160 ----HEPSTDAYETYDRQQSAVKVIMNCFYGVLGWDRFRLYDKEMGAAVTATGRDVIEFTEQAAA-ELD-------------------------------------KDVIYGDTDSVMLEL---------GNEVSEDEAIEQSFEIESHINDAYDRFAERLNAEE--HRFQIEFEKLYRRFFQ--------AGKKKRYAG-------HIIWKE---------------GKEVDDLDITGFEYQRS

15789745 E Halobacterium_sp-_NRC-1 ----HEPDSEDYERYDRQQAAVKVIMNSLYGVFGWDRFRLYDRAMSAGVTSTNREVIDFTEQAAE-EFG-------------------------------------YEVAYGDTDSVMLEL---------GDDMTKAEAIAESFDIEDHINAAYDEFAREQLHADD-HRFQIEFEKLYRRFFQ--------AGKKKRYAG-------HIVWKE---------------GKDVDDVDITGFEYQRS

88602445 E Methanospirillum_hungatei_JF-1 ----YPFGSQEYQLYDMQQNVIKVIMNTYYGVSGYSRFRLYDREIGSAVTSVGRAIIEHTRNTIE-NMG-------------------------------------YSVIYGDTDSCMIQI----------PATSLEETITKAREIEAVLNESYNTFAREVLHAEK-HYFSIKFEKVYRRFFQ--------GGKKKRYAG-------NLIWKE---------------GKSVDETDMVGFEAKRS

154150651 C Candidatus_Methanoregula_boonei_6A8 ----FPYGSAQYVLYDMQQNVLKVIMNTYYGVSGYTRFRLFDREIGAAVTSVGRAIIEHTRHVIE-QEG-------------------------------------YKVIYGDTDSCMVQI----------PPLDREKTIETARTLEKKLNTSYQDFARNVLNADT-HYFSIKFEKIYARFFQ--------AGKKKRYAG-------RLVWKE---------------GKEADQIDIVGFEIRRS

126178748 E Methanoculleus_marisnigri_JR1 ----YPFGSPEYVLYDLQQNVLKVIMNSYYGVSGYTRFRLYDREIGSAVTSVGRAIIRHTRDIIT-NLG-------------------------------------YTVLYGDTDSCMIEV----------PPGDLEATIARAREIEAKLNASYGDFAKTELNADT-HYFSIKFEKVYRRFFQ--------AGKKKRYAG-------HLVWKE---------------GKDVDEVDVVGFEIRRS

124485441 E Methanocorpusculum_labreanum_Z ----FPFGSDEYTLYDMQQNVLKVIMNTYYGVSGFSRFRLYDRDIGAAVTSVGRAIIQHTKLVIT-KRG-------------------------------------YDVIYGDTDSCFVQI----------PFTSLEDTMKIAREIEEELNASYLSFARETLGADM-NFFSIKFEKIYRRFFQ--------GGAKKRYAG-------HLIWKE---------------GQDVDKIDITGFEMKRS

116754694 E Methanosaeta_thermophila_PT ------AGEDERAFLDAKQYALKILLNSFYGYSGYARARLYSLTLANAVTSFGRHNILRTKEMIE-EIGSVYIVDGKALLPEETSVSSNPKPPDTIELNSAGRRYDLSVVYGDTDSVFVRI-------SSGYSITPEDAELIGRKIAETITSKLP------------KPMELVFEAFARRAIF---------LAKKRYALWLFERVSTQSKPLDGVEGSSKTPSEGSGIIWRDRIKVRGMETVRR

91773640 E Methanococcoides_burtonii_DSM_6242 -----ASDEGEYQVLDATQLALKILLNSFYGYSGYARARLYSLQMANSVTSIGRGNIARTQDIVCNRIGTVILRDNKAYLRDEVDELKDDDKAVK-----------LSVAYGDTDSVFVHCKNDQDLEFEEGVFSLEDSALVGKKVASIVTASLP------------DPMELEFEATAKRVLL---------IAKKRYAQWIFEPA-NGGWTD--------------------SIKVKGLETVRR

21228106 E Methanosarcina_mazei_Go1 -----TSDKNEYRVLDATQLAIKILLNSFYGYSGYARARLYSLTLANAVTSFGRSNILNTRDLINNDIGKIVLRDSAALFLEEAGEISPQDRVID-----------LSVAYGDTDSVFVHC-------RSEGDLSLEDVSLVGNRLSQIVSASLP------------DPMELEFEAIAKRALL---------IAKKRYALWLFEPR-NSGWEN--------------------KIKVKGMETVRR

41614864 N Nanoarchaeum_equitans_Kin4-M ----LDRDSEEYKIINAKQAVLKIIINATYGYMGFPNARWYCIDCAAAVAAWGRKYINYILKRAE-EEG-------------------------------------FKVIYGDS-----------------------IMDTEIEVIENGIKKK--------------EKLSDLFNKYYAGFQI----------GEKHYAF---------------------------------PPDLYVYDGERW

84490287 E Methanosphaera_stadtmanae_DSM_3091 -----ETVPEQKKAYDFEQQGLKRLANSMFGAYGYARFRWYKKECAAAITAWGREYIQDAMKKSE-EYG-------------------------------------FKPIYADTDGFYATY----------------------------------------------------------------------------------------------------------------------------

15679219 E Methanothermobacter_thermautotrophicus -----SDDPMERKILNVQQEALKRLANTMYGVYGYSRFRWYSMECAEAITAWGRDYIKKTIKTAE-EFG-------------------------------------FHTVYADTDGFYATY----------------------------------------------------------------------------------------------------------------------------

15669075# E Methanocaldococcus_jannaschii_DSM_2661 -MAEIGEINEEYNLLDYEQKSLKILANSIYGYLAFPRARFYSRECAEIVTYLGRKYILETVKEAE-KFG-------------------------------------FKVLYIDTDGFYAIW---------KEKISKEELIKKAMEFVEYINSKLP------------GTMELEFEGYFKRGIF---------VTKKRYA-----------LIDE-----------------NGRVTVKGLEFVRR

45357943 E Methanococcus_maripaludis_S2 -KAEKGEFDEEYQILDYEQRSIKVLANSHYGYLAFPMARWYSRDCAEITTHLGRQYIQKTIEEAE-NFG-------------------------------------FKVIYADTDGFYSKW------ADDKEKLSKYELLEKTREFLKNINNTLP------------GEMELEFEGYFKRGIF---------VTKKKYA-----------LIDE-----------------NEKITVKGLEVVRR

57639936# E Thermococcus_kodakarensis_KOD1 -----TIDPIERKLLDYRQRAIKILANSIYGYYGYARARWYCKECAESVTAWGREYITMTIKEIEEKYG-------------------------------------FKVIYSDTDGFFATI----------PGADAETVKKKAMEFLKYINAKLP------------GALELEYEGFYKRGFF---------VTKKKYA-----------VIDE-----------------EGKITTRGLEIVRR

6015025 E Thermococcus_gorgonarius -----TIDPIEKKLLDYRQRAIKILANSFYGYYGYAKARWYCKECAESVTAWGRQYIETTIREIEEKFG-------------------------------------FKVLYADTDGFFATI----------PGADAETVKKKAKEFLDYINAKLP------------GLLELEYEGFYKRGFF---------VTKKKYA-----------VIDE-----------------EDKITTRGLEIVRR

14521919 E Pyrococcus_abyssi_GE5 -----SKDPVEKKLLDYRQRAIKILANSYYGYYGYAKARWYCKECAESVTAWGRQYIDLVRRELE-SRG-------------------------------------FKVLYIDTDGLYATI----------PGAKHEEIKEKALKFVEYINSKLP------------GLLELEYEGFYARGFF---------VTKKKYA-----------LIDE-----------------EGKIVTRGLEIVRR

11498108 E Archaeoglobus_fulgidus_DSM_4304 ----LSPESSEYKLLDIKQQTLKVLTNSFYGYMGWNLARWYCHPCAEATTAWGRHFIRTSAKIAE-SMG-------------------------------------FKVLYGDTDSIFVTK--------------AGMTKEDVDRLIDKLHEELP--------------IQIEVDEYYSAIFF---------VEKKRYAG----------LTED------------------GRLVVKGLEVRRG

20094475 E Methanopyrus_kandleri_AV19 --LDTESHPHEAKILDVRQQAYKVLANSYYGYMGWANARWFCRECAESVTAWGRYYISEVRRIAEEKYG-------------------------------------LKVVYGDTDSLFVKL----------PDADLEETIERVKEFLKEVNGRLP--------------VELELEDAYKRILF---------VTKKKYAG----------YTED------------------GKIVTKGLEVVRR

15897046 C Sulfolobus_solfataricus_P2 -----------------------ELISSFNDYVHWVNARWYSREIASAFDEFSNEIIRFIIDLIK-SSG-------------------------------------LDVILANDLLIFVTG----------------GSRDKVNELITKINSLYN--------------LDVKVKIFYKSLLV---------LDNNRYAG----------LSEG------------------DKIDIARKGEEDM

146304901 C Metallosphaera_sedula_DSM_5348 -----AKDEYEKRRLDERQRALKIMANAMYGYMGWLGARWYSKEGAEAVTAWGRQTIMTAAEIAK-NSG-------------------------------------FEVIYGDTDSIFVKG-----------------DMSSVELLTQKIVQALD--------------LDIKVDKKYKKVFF--------TENKKRYAG----------LTFD------------------GKIDIVGFEAIRG

159041906 C Caldivirga_maquilingensis_IC-167 ----YPEGSFEWVLLNERQRALKIMANAMYGYCGWLGARWYIREVAESVTAWGRYLLKTAMSMAK-ERG-------------------------------------LTVIYGDTDSLFVTY-----------------DKDKVADIISRINEM--------------G-FEVKIDKVYSKLIF--------TESKKRYIG----------LTAD------------------GEVDIVGFEAVRG

119719310 C Thermofilum_pendens_Hrk_5 ----LDPRSEEYRIYDERQKAIKVITNATYGYSGWSMARWYKREVAEATTAWGRELIKATIKKAQ-SLG-------------------------------------LSIIYGDTDSIFVQF-----------------DEEKIGRLVEYVEKEL-------------G-FEIKLDKVYEKVFF--------TESKKKYCG----------LLAD------------------GRVDLVGFEAVRG

126465787 C Staphylothermus_marinus_F1 ----YPPTSYEYRLLDERQKAVKVLANATYGYMGWIHARWYCRECAEAVTAWGRQTIKSAIELAR-KLG-------------------------------------LKVIYGDTDSLFVTY-----------------DKDKVEKLIELIQTKL-------------G-FEIKIDKIYKRVFF--------TEAKKRYAG----------LLED------------------GRIDIVGFEAVRG

124027770 C Hyperthermus_butylicus_DSM_5456 ----LPPESPEYRLYDERQRALKVLANASYGYMGWSGARWYCKRCAEAVTAWGRSLILSAIEYAR-KLG-------------------------------------LKVIYGDTDSLFVTY-----------------DPEKVKKLIDYVVNVL-------------G-FEIKIDKIYRRVFF--------TEAKKRYVG----------LLED------------------GRIDIVGFEAVRG

156936857 C Ignicoccus_hospitalis_KIN4-I ----LSPEDPLYKLLDNRQKAVKVLANAHYGYMGWPHARWYCRECAEAVTSWGRELILKAIQMAR-ELG-------------------------------------LKVIYGDTDSLFVIY-----------------DKEKVEKLIERIEKEL-------------G-FEIKIDKIYKKLFF--------TEAKKRYAG----------ILED------------------GRIDVVGFEAVRG

118431730 C Aeropyrum_pernix_K1 ----HPPDSPEYKLLDERQKAIKLLANASYGYMGWPHARWYCRECAEAVTAWGRSIIRTAIRKAG-ELG-------------------------------------LEVIYGDTDSLFVKN-----------------DPEKVERLIRFVEEEL-------------G-FDIKVDKVYRRVFF--------TEAKKRYVG----------LTVD------------------GKIDVVGFEAVRG

18313103 C Pyrobaculum_aerophilum_str-_IM2 ----YPPDSPEYRVLDERQRALKIMANAMYGYTGWVGARWYKKEVAESVTAFARAILKDVIEYAR-KAG-------------------------------------IVVIYGDTDSLFVKK------------------SGDVEKLVKYVEEKY-------------G-IDIKIDKDYSTVLF--------TEAKKRYAG----------LLRD------------------GRIDIVGFEVVRG

170290810 K Korarchaeum_cryptofilum_OPF8 ----LKQDDPEYKRLDAAQRAIKVVTNAIYGYMGWESATFMNISAARLTSAYGRLYIKKVRGMLE-SKG-------------------------------------LSVIYIDTDGIQFLG-------------------KECENVIDDINSGIP--------------LRLDLRYKALRGIY--------LAKKK-YAH----------LLED------------------GRIIAKGFEFVRR

**E-exonuclase; P-polymerase; N-nucleotide binding ......................NNNNNNNNNNNN............................................................................P.PPPP.................................................................................................................................**

170290793 K Korarchaeum_cryptofilum_OPF8 ----EEKDENMREWFDTVQRSLKVFLNASYGVFGYENFPLYSPPAAEMITALARKAMLLSIDEAR-RMN-------------------------------------LSVIYGDTDSLFIRG----------------ATQGQIEEFERRVEEKL-------------G-IDLELDKWYRYVVF--------SRLKKNYLG----------VTKD------------------GSVVIKGLLGKKR

14600448 C Aeropyrum_pernix_K1 ---DKSLREEERLWYDVVQSAMKVYINASYGVFGSEKFSLYSLPVAESVTALGRAVLRGTLEKSR-ELN-------------------------------------LHIVYGDTDSLFIWD----------------PPKDVLNDLVDYVERTY-------------G-LELELDKVFRAILF--------SGLKKNYLG----------ITEE------------------GDIVIKGMVAKKS

146304655 C Metallosphaera_sedula_DSM_5348 ---QSNIDEERKMLYDVVQRGMKVFINATYGVFGAETFPLYAPAVAESVTALGRYVITSTKEMAN-KLG-------------------------------------LKVVYGDTDSLFIHQ----------------PDKKKLEELVEWTRQNF-------------G-LDLEVDKTYRFIAF--------SGLKKNYFG----------VFKD------------------SKVDIKGMLAKKR

15897474 C Sulfolobus_solfataricus_P2 (1S5J 190-686) ---NPNNSEEQKLLYDVVQRAMKVFINATYGVFGAETFPLYAPAVAESVTALGRYVITSTVKKAR-EEG-------------------------------------LTVLYGDTDSLFLLN----------------PPKNSLENIIKWVKTTF-------------N-LDLEVDKTYKFVAF--------SGLKKNYFG----------VYQD------------------GKVDIKGMLVKKR

**Secondary structure (1S5J) E-β-strand; H-α-helix HHHHHHHHHHHHHHHHHHHHHHHHHH HHHHHHHHHHHHHHHHHHHHHHH EEEEE EEEEE HHHHHHHHHHHHHH EEEEEEEEEEEE EEE E EE**

126465771 C Staphylothermus_marinus_F1 ---SKEIPIEMRAWYDVVQKAMKVFINASYGVFGAENFPLYSPAVAESVTALGRKSLYSILKKSA-EIG-------------------------------------MKVVYGDTDSIFLWA----------------PTNEQLTKLQEWVSKNL-------------G-LEIEVDKEFVYVLF--------TGLKKNYIG----------RYVN------------------GGIEIKGLMAKKR

156937483 C Ignicoccus_hospitalis_KIN4-I ---DKSLDDKMKMWYDTVQSAMKVYINASYGVLGAESFELYCPPAAESITAYGRFAIKSTMDYAK-KNK-------------------------------------IAVLYGDTDSMFLWD----------------PPQNLLDDIIEWVKNNF-------------G-LEIEIDKTYRFVAF--------TGLKKNYIG----------VYPG------------------GEIDVKGLLGKKR

124028129 C Hyperthermus_butylicus_DSM_5456 ---DKSLPEDVRAWYDTVQAAMKVYINASYGVFGASSFPLYAPPVAESVTAIGRYTIKETVKKAA-ELG-------------------------------------LRVLYGDTDSLFIWN----------------PDEEKLEELQKYVEENF-------------G-LDLEVDKVYKFVTF--------SGLKKNYIG----------AYED------------------GSVDVKGMVAKKR

159040732 C Caldivirga_maquilingensis_IC-167 ----GDKDPRQRDYYNVVQAALKVFINASYGVFGAEIFPLYCPPLAELVTALGRLAITRTIIKAL-DLG-------------------------------------LTPIYGDTDSLFLYN----------------PSKDKLEEMINWVKDEM-------------G-IDIELDKAYRILAL--------SGRKKNYAG----------ILQD------------------GSVDMKGLVGKKR

171185814 C Thermoproteus_neutrophilus_V24Sta ----KAPTPAERQLYDVVQSAMKVFINASYGVFGAETFPLYCPPVAELTTALARYIMTSTVLKAI-ELG-------------------------------------MIPVYGDTDSLFLWN----------------VTEDKIKRLVQYA-EEI-------------G-IDIELDKIYKFVMF--------SGRKKNYLG----------VTND------------------GSVIVKGIVAKKR

18313158 C Pyrobaculum_aerophilum_str-_IM2 ----KAPTPAERQLYDVVQSAMKVFINASYGVFGAETFPLYCPPVAELTTALARYIMTSTVLKAI-ELG-------------------------------------LIPVYGDTDSLFLWN----------------VSEEKLKKLVEYTEEL--------------G-IDIELDKIYKFVMF--------SGRKKNYLG----------VTND------------------GSVIVKGIVAKKR

119719216 C Thermofilum_pendens_Hrk_5 ----SSKNEEERQQYDVIQSALKVFLNASYGVFGSEAFPLYCPPVAESTTALGRYSILKTMQKAV-EMG-------------------------------------IPVLYGDTDSLFLWN----------------PPQEKLEELLQNILNEL-------------Q-IDLSIDKEYLWVVF--------SKRKKNYLG----------LLKE------------------RKVDIKGLTGKKR

161528456 C Nitrosopumilus_maritimus_SCM1 ---KETLTDEQRQQYTVVSQALKVILNASYGVMGAEIFPLYFLPAAEATTAIGRHTILETIKKCE-SAG-------------------------------------IEVLYGDTDSLFIKN----------------PTKEQIQIVIDQAKKDH-------------G-VDLEIDKTYRYCVL--------SNRKKNYLG----------VTKE------------------GKVDVKGLTGKKS

3599383 C Cenarchaeum_symbiosum ---SQSITEEQRQQYTVISQALKVVLNASYGVMGAEIFPLYFLPAAEATTAVGRYIIMQTISHCE-QMG-------------------------------------VKVLYGDTDSLFIKN----------------PEERQIHDIVEHAKKEH-------------G-VELEVDKEYRYVVL--------SNRKKNYFG----------VTKS------------------GKVDVKGLTGKKS

188997124 B Sulfurihydrogenibium_sp ---------EKNQIYDNRQKALKWLLVVSFGYLGYKNAVFGRIESHEATTSIGRQLITFVKEILE-AKG-------------------------------------FRVIHILTDSIWVYK--------------HDYTIDDYKKMEEYLNKRINEKFIPVNP-DGIP-FKILLEGVYDWIVFLPSKS-NSVGVSNRYFG----------KFSN------------------GEFKFRGIDLRRR

183221542 B Leptospira_biflexa_serovar- ----KRNNHPNLLNIQSKQSSLKWMLVTSFGYLGYRNAKFGKLESHEAVTAFGREKLINAKEVSE-EFQ-------------------------------------YKVVHGITDSIFIQK---------------KDTTPITNEDLTFLCLEIE---------KRTK-IKMEIEGIYSWLCFPPSTQDEKLPVANRYMG----------RFID------------------GQFKGRGIITRRK

124515762 B Leptospirillum_sp-_Group_II_UBA ----RSVPDKKTDPDNLRQKALKWLLVVSFGYLGYKNARFGRIEAHECVTAYGREVLLKAKEMAE-DRG-------------------------------------FRVLHGIVDSLWLQK------------------QGMTQDVCEELAREIG---------KETG-IPLNLEGIYRWIGFFSSRTSPSIAVPNRFLG----------VFET------------------GEIKVRGLEIRRK

16120259 E Halobacterium_sp-_NRC-1 ETDRDDPDEDRLAELEGRSGALKWILVACFGYQGFSNAKFGRIECHEAINAFAREILLMAKQRLE-AGG-------------------------------------WRVVHGIVDSIWVTP----------DPDVDEEDREDLQTLATEITERV-------------E-IRLEHEAHYDWVAFVPQRE-SDAGALTKYFG----------KVAG-----------------DDDFKVRGIEARQR

11500019 E Archaeoglobus_fulgidus_DSM_4304 ---------RKDSRYAGVDSILKWMLVTCFGYTGYRNAKFGRIEVHEEINRIGREILIKTKEIAE-ELG-------------------------------------FKVLHGIVDCLWVRG----------------------EDIGSFQARVE----------EETG-LLTEVEK-FDWIVFLPMKD--GSGAYNRYYG----------RLSS------------------GEMKLRGVMARRR

88602132 E Methanospirillum_hungatei_JF-1 -----------DPSYAGMDGVLKWMLVTCFGYTGYKNARFGRIEVHEEITRAATRILKDCVKLTE-AHG-------------------------------------GKVLHAIIDCLFIRG-------------------GHAGTIQEEIERMT-------------T-FHTE-ADTYDWVVFLPQSD--GSGSYCSYFG----------RLKT------------------GKMKVKGVSTNRR

154150796 E Methanoregula_boonei_6A8 -----------DPRLRGPDAILKWMLVTCFGYTGYRNAKFGRIEVHEAITAASREILLRTKEIAE-EMG-------------------------------------FTVLHGIVDCLWVQG------------------NLPIETLRERIERET-------------R-YPID-AEHFDWIVFLPLAD--GFGAYNRYYG----------KLSD------------------GHIRVRGIAARRH

16081572 E Thermoplasma_acidophilum_DSM_1728 -----------SVVYHSRNVALKWLLLTSFGYTGYKNAKFGKIEVHERITELGRKALAEAIAVAH-ENG-------------------------------------FEMIHGIVDSLWIRG------------------SGSIERVVSEISRRT-------------R-IDIVVSGHYHWIAFLPERD--GTGSPSRYIG----------LDTS------------------GKYKIRGLMIRRS

126008334 E Ferroplasma_acidarmanus_fer1 -----------SDIYASRNKALKILLLNSFGYTGYKNAKFGRIDVHEKITGIGRKIIADSMRISE-KNG-------------------------------------FNVLHGVVDSLWLSG------------------NGDIEKTLKEIYEKT-------------G-IPIVLDSNYRWIAFMPKAN--GIGSANRYIG----------LRKD------------------GTFKVRGIELRRR

126465179 C Staphylothermus_marinus_F1 ---------TNNEIYSLRSKALKWILVSGFGYLGFKNSLFGSIMAHETVTWYARRILREAHRLLE-NRG-------------------------------------YKVIHIIIDSLFVQG-------------------GDCEKALRIVEET--------------G-MPAKIEAEYTWLYIPKTKN-TGLGASNRYYG----------RLVS------------------GEMKIKGVMCVRK

118431787 C Aeropyrum_pernix_K1 ----------------EEQEAIKWILVSGFGYLGFRNSLFGSIAAYETVTAAARRALAAAEEAAV-EMG-------------------------------------YRVIHSLVDSVFIQP---------------VEPRGPPGEVAREIERRT-------------G-VPVKIEAEYKWLYIPPTLR--GHGAVNKYYG----------ALES------------------GGVKLKGIAAVRR

146304209 C Metallosphaera_sedula_DSM_5348 -----------------RAEAIKWILVASFGYLGYRNSKFGKIEAYELVTYYARKTLRRALEIAQ-EIG-------------------------------------VEVLHGIVDSLVIRG--------------------DAPQLVRRLEEET-------------G-LHLR-STRLKWIVLGGRRD--GLPYPMRYFG----------MTE-------------------EGMKYKGI--IRR

15898291 C Sulfolobus_solfataricus_P2 -----------------RAEAIKWILVASFGYLGYRNSLFGKIEAYEMVTYLARKTLRRTMEIAE-EMG-------------------------------------LRVLHGIIDSLVVKG-------------------DNVDKFIEKVEKET-------------G-LRLD-YKRYNWIIFTITRN--NTPYPTRYIA----------NMN-------------------GEMIAKGL--IRE

The order of the sequences is the same as in the phylogenetic tree (**Figure 1**). Elements of secondary structure are shown in blue for the Sulfolobus solfataricus polymeras (PDB 1S5J (Savino, Federici et al. 2004). Motifs implicated in the endonuclease and polymerase acitivties and in nucleotide-binding are shown in red and designated accordingly. Gray letters indicate regions with low confidence in alignment. Symbol # marks sequences for which intein-containing regions were removed from the alignment

Savino, C., L. Federici, et al. (2004). "Insights into DNA replication: the crystal structure of DNA polymerase B1 from the archaeon Sulfolobus solfataricus." Structur**e** 12(11): 2001-8.
